# An Ensemble Learning-Based Method for Inferring Drug-Target Interactions Combining Protein Sequences and Drug Fingerprints

**DOI:** 10.1155/2021/9933873

**Published:** 2021-04-24

**Authors:** Zheng-Yang Zhao, Wen-Zhun Huang, Xin-Ke Zhan, Jie Pan, Yu-An Huang, Shan-Wen Zhang, Chang-Qing Yu

**Affiliations:** School of Information Engineering, Xijing University, Xi'an 710123, China

## Abstract

Identifying the interactions of the drug-target is central to the cognate areas including drug discovery and drug reposition. Although the high-throughput biotechnologies have made tremendous progress, the indispensable clinical trials remain to be expensive, laborious, and intricate. Therefore, a convenient and reliable computer-aided method has become the focus on inferring drug-target interactions (DTIs). In this research, we propose a novel computational model integrating a pyramid histogram of oriented gradients (PHOG), Position-Specific Scoring Matrix (PSSM), and rotation forest (RF) classifier for identifying DTIs. Specifically, protein primary sequences are first converted into PSSMs to describe the potential biological evolution information. After that, PHOG is employed to mine the highly representative features of PSSM from multiple pyramid levels, and the complete describers of drug-target pairs are generated by combining the molecular substructure fingerprints and PHOG features. Finally, we feed the complete describers into the RF classifier for effective prediction. The experiments of 5-fold Cross-Validations (CV) yield mean accuracies of 88.96%, 86.37%, 82.88%, and 76.92% on four golden standard data sets (*enzyme*, *ion channel*, *G protein-coupled receptors* (*GPCRs*), and *nuclear receptor*, respectively). Moreover, the paper also conducts the state-of-art light gradient boosting machine (LGBM) and support vector machine (SVM) to further verify the performance of the proposed model. The experimental outcomes substantiate that the established model is feasible and reliable to predict DTIs. There is an excellent prospect that our model is capable of predicting DTIs as an efficient tool on a large scale.

## 1. Introduction

The identification of interacting drug-target pairs is of cardinal significance in pharmaceutical science. Previous development of genomics, protein engineering, and molecular biology dynamically helps researchers in finding the potential therapeutic drugs and explaining the by-effect of a trial. In past decades, the Food and Drug Administration (FDA) declared that the demand for new drugs is hard to meet due to the adverse clinical outcomes of some candidate drugs [1]. Classifying DTIs remains to be a critical step for better developing and applying novel molecule-targeted drugs. Previously, researchers utilized clinical experiments as the main approach to discover DTIs. Nevertheless, the traditional experiments are still cumbersome, costly, and time-consuming. Meanwhile, it also has to confront the contingency and inefficiency of the results. Therefore, novel computer-aided drug development (CADD) methods need to be advanced for effectively avoiding these drawbacks [2].

With the progress of protein primary sequence detection technologies and spectral techniques in determination of the chemical composition structure of drugs, the public database has had an explosive growth in size. These databases which provide multiple download formats comprehensively construct a reliable data platform for researchers. Different kinds of databases, such as the Therapeutic Target Database (TTD) [3], DrugBank [4], ChEMBL [5], and KEGG [6], collect the information of the protein primary structure, drug molecular structure, and drug-target pairs with known interactions to assist establishing the prediction model of DTIs. In the past years, researchers have made many achievements in predicting DTIs by combining traditional computing methods and bioinformatics. The most widespread applications are based on molecular docking, genome, and pharmacophore [7]. Molecular docking simulation is utilized to detect the optimal binding position between drug molecules and targets based on energy matching. This method also requires complete three-dimensional (3D) substructures of proteins, but they are hard to explore by Nuclear Magnetic Resonance (NMR), electron microscopy, and X-ray crystallography [8]. Pharmacophores are a characteristic element of drug-active molecules that play a pivotal role in the prediction of DTIs [9]. Researches suggested that the pharmacophore method can effectively inspect the multitarget drug design and reduce the blindness of screening. The difficulty of matching molecular pharmacophores is determined by the number of pharmacophore characteristics. In addition, whether the molecule can match the pharmacophore is also related to the conformations of the molecules [10]. When the conformation changes, the molecule will not match the existing pharmacophore model. Therefore, the establishment of the pharmacophore model is still not comprehensive for further bioassay. At the same time, this method does not take 3D structures of targets into account, which declines the accuracy of the pharmacophore model [11]. In general, it is exceedingly urgent to develop more robust and universal methods for the prediction of DTIs without a ligand and 3D target structure.

Up to now, many learning-based models are developed to detect potential DTIs. For instance, Ding et al. [12] developed a fuzzy bipartite local model (FBLM) based on fuzzy least square support vector machine and multiple kernel learning (MKL) for predicting DTIs. Specifically, MKL is employed to fuse multiple kernels of drugs and targets, and FBLM is adopted to infer the unknown DTIs. Krisztian et al. [13] utilized the Modified Linear Regression (MOLIERE) to predict the potential DTIs based on asymmetric loss models (ALM). Ye et al. [14] proposed a new prediction framework based on Adversarial Bayesian Personalized Ranking (AdvB). More specially, the latent factor matrices of drugs and targets are trained by partial order relationships. Then, the scores of inner products of factors are trained to predict DTIs. Maryam et al. [15] developed an effective model named the Coupled Tensor-Matrix Completion (CTMC) to repurpose drug molecules by constructing drug-drug and target-target tensors. Pliakos and Vens [16] proposed to address DTI prediction as a multioutput prediction task by learning ensembles of multioutput biclustering trees (eBICT) on reconstructed networks. An et al. [17] combined Weighted Extreme Learning Machine (WELM) and Speed Up Robot Features (SURF) to predict DTIs. Laarhoven et al. [18] proposed the Kronecker Regularized Least Square- (Kron-RLS-) based predictive models, which employed the Kronecker product to fuse drug and target feature spaces. Gönen [19] proposed a joint Bayesian formulation of projecting drug compounds and target proteins into a unified subspace, and this formulation combines dimensionality reduction, matrix factorization, and binary classification for predicting drug-target interaction networks. Zheng et al. [20] proposed a factor model, named Multiple Similarity Collaborative Matrix Factorization (MSCMF) which is an extension of weighted low-rank approximation for one-class collaborative filtering. In this model, drugs and proteins are projected onto low dimensional feature space, and the weights of low-rank matrix and similarity matrix are estimated by alternating least square method to predict DTIs.

**Figure 1:**
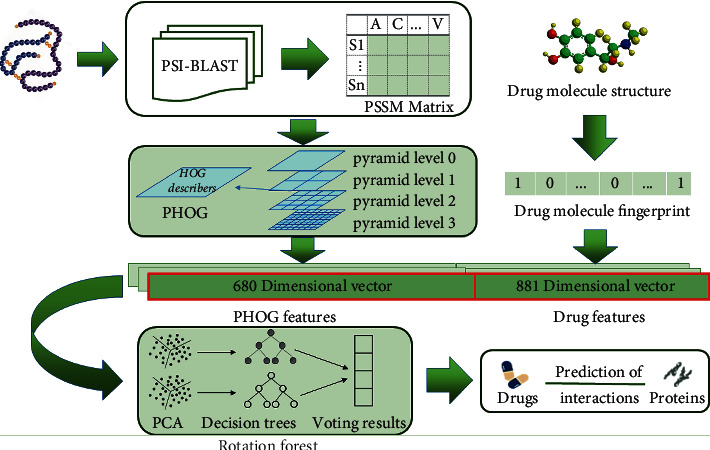
Workflow for the proposed model to predict DTIs.

In this study, we present a novel computational method which exploits protein primary sequence and molecular fingerprints of drug compounds. More specially, this model numerically characterizes different amino acids as PSSMs to carry biological evolution information. Then, the proposed model employs the PHOG approach to extract the 680-dimensional local features of PSSM from different pyramid levels. Finally, the RF classifier is employed to effectively predict DTIs based on the fusion which contains PHOG descriptors of PSSMs and drug fingerprints. This experiment also evaluates the prediction performance by conducting 5-fold Cross-Validation (CV) on *enzyme*, *ion channel*, *G protein-coupled receptors* (*GPCRs*), and *nuclear receptor* data sets. For the sake of verifying the reliability of the model, we also carried out the state-of-the-art LGBM and SVM on benchmark data sets. The overall results of the experiments illustrate that the established model is practicable in providing accurate candidates for clinical experiments by predicting DTIs.  Figure 1 depicts the workflow of the proposed model.

## 2. Materials and Methods

### 2.1. Data Sets

In this paper, entire experiments were performed on benchmark data sets, viz., *enzyme*, *ion channel*, *GPCRs*, and *nuclear receptor*. All data sets originate from the databases of DrugBank [4], SuperTarget [21], BRENDA [22], and KEGG BRITE [6]. The statistical quantities of existing drugs are 445, 233, 210, and 54, respectively. The numbers of known proteins are 664, 95, 204, and 26, respectively. The counts of the DTIs which have been proven are 2926, 635, 1476, and 90, respectively. The number of known DTIs which were regarded as positive sample data set is 5127.  Table 1 fully lists the statistical amounts of drugs, target proteins, and DTIs.

In this section, the bipartite graph is employed to display the DTI network. The nodes of the graph denote drugs and proteins, the edges which connect the nodes denote the relationships between drugs and targets. The interacting drug-target pairs are considered as positive samples; the others are regarded as negative samples in the sparse network. Taking the *ion channel* data set as an instance, there are 42840 (210 × 204) edges existing in the graph. The verified 1476 real drug-target interactions construct the positive sample set, and the residual 41364 (42840-1476) pairs represent the negative samples. It is obvious that there is a big quantity gap between positive samples and negative samples. For attaining sample balance, a downsampling algorithm is adopted in uncorrelated pairs to form the negative set which contains the same number of samples as the positive one. In consideration of the scale of the sparse network and the large ratio of differences, the possibility that the drug-target pairs with real interactions are collected in the negative data set can be ignored. Therefore, the sample quantities of four negative data sets are 2926, 1476, 635, and 90, respectively.

### 2.2. Drug Substructure Characterization

In recent years, many physical and chemical properties are utilized to describe the drug compound information including geometry, topology, and quantum chemistry [23, 24]. At present, the researchers demonstrate that molecular fingerprints can effectively characterize the drug substructure. The fingerprints of structural bonds represent the drugs as Boolean substructure vectors by separating the drug molecular structure into a variety of segments. Although the molecule is sliced into individual segments, it still retains the entire structure information of the drug [25, 26]. These printers reduce the information loss and error accumulation in the process of description and screening. Specifically, the predefined dictionary which contains all substructures matches all fragments of the given drug molecule. If the fragment exists in the dictionary, the corresponding position in the carrier is set to 1; otherwise, it is set to 0. The complete fingerprint database provides an effective way to describe the molecular structure of drugs as binary fingerprint vectors. We utilized the chemical structure map from the PubChem system in the website https://pubchem.ncbi.nlm.nih.gov/, and the map contains 881 molecular substructures [27]. Hence, the feature describers of the drug molecular structure take the form of an 881-dimensional binary vector.

### 2.3. Position-Specific Scoring Matrix (PSSM)

In general, researchers took many physicochemical approaches to numerically describe target proteins [28]. The effective descriptors will differentially convert proteins to enhance the performance of the classifier. Within the experiment, the Position-Specific Scoring Matrix (PSSM) is utilized to represent the biological evolution of proteins [29], and this matrix contains the probability information of 20 amino acids at each position in the original protein sequence. In the practical process, the Position-Specific Iterated Basic Local Alignment Search Tool (PSI-BLAST) is employed to generate the corresponding PSSM for different sorts of amino acids. The matrix is as follows:

(1) $$\begin{array}{c} \text{PSSM}=\left[\begin{array}{cccc} \ell_{1,1} & \ell_{1,2} & \cdots & \ell_{1,20}\\ \ell_{2,1} & \ell_{2,2} & \cdots & \ell_{2,20}\\ \vdots & \vdots & \ddots & \vdots \\ \ell_{n,1} & \ell_{n,2} & \cdots & \ell_{n,20} \end{array}\right], \end{array}$$

where the PSSM is expressed as a matrix of *L* × 20 and *L* denotes the length of the amino acid. *ℓ*_*i*,*j*_ denotes the evolutionary score that the *i*_th_ residue mutates into the *j*_th_ amino acid in the evolutionary process. The experiments also optimized the parameters of PSI-BLAST to obtain more reliable homologous sequences. In summary, parameter *e* which represents the noise of protein matching is assigned to 0.001, and the frequency of iterations is set to 3.

### 2.4. Pyramid Histogram of Oriented Gradients

The pyramid histogram of oriented gradients (PHOG) is a feature extraction method which describes the local features by counting the distribution of the gradient direction histogram from different pyramid levels [30]. Meanwhile, this method has strong antinoise performance and antirotation ability [31]. Firstly, the given original image *F* is segmented into *i* × *i* spatial grids in the *i*_th_ pyramid level. Then, the histogram of oriented gradient (HOG) vectors of each grid should be calculated. Herein, we adopted Sobel operators to detect the edges and reduce the noise of the image. The Sobel operators can be defined as follows:

(2) $$\begin{array}{c} {\text{Sobel}}_{x}=\left[\begin{array}{c} 1\\ 0\\ -1 \end{array}\right]\times \left[1\;2\;1\right]=\left[\begin{array}{ccc} 1 & 2 & 1\\ 0 & 0 & 0\\ -1 & -2 & -1 \end{array}\right], \end{array}$$

(3) $$\begin{array}{c} {\text{Sobel}}_{y}=\left[\begin{array}{c} 1\\ 2\\ 1 \end{array}\right]\times \left[\begin{array}{ccc} 1 & 0 & -1 \end{array}\right]=\left[\begin{array}{ccc} 1 & 0 & -1\\ 2 & 0 & -2\\ 1 & 0 & -1 \end{array}\right], \end{array}$$

where Sobel_*x*_ and Sobel_y_ represent the horizontal operator and the vertical operator individually [32]. Then, the first-order differential Sobel operator is utilized to convolute the given image as follows:

(4) $$\begin{array}{c} G_{x}=F*{\text{Sobel}}_{x}, \end{array}$$

(5) $$\begin{array}{c} G_{y}=F*{\text{Sobel}}_{y}, \end{array}$$

where *G*_*x*_ denotes the convolution of picture *F* in the *x*-axis direction, where *G*_*y*_ denotes the convolution of image *F* in the *y*-axis direction. After convolution, the image *F* is converted into *I* which can be obtained as follow:

(6) $$\begin{array}{c} I=\sqrt{G_{x}^{2}+G_{y}^{2}}. \end{array}$$

**Figure 2:**
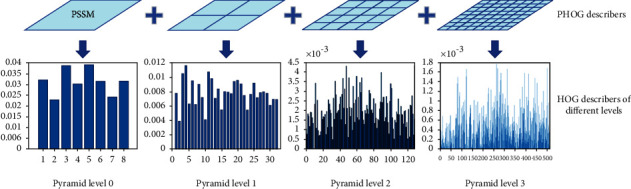
The example of merging HOG describers into PHOG describers.

The gradient magnitude *g* and direction *θ* of pixels in grids can be obtained by the following formulas:

(7) $$\begin{array}{c} g\left(\varphi ,\omega \right)=\sqrt{{\text{g}}_{x}{\left(\varphi ,\omega \right)}^{2}+g_{y}{\left(\varphi ,\omega \right)}^{2}}, \end{array}$$

(8) $$\begin{array}{c} \theta \left(\varphi ,\omega \right)=\arctan\frac{g_{y}\left(\varphi ,\omega \right)}{g_{x}\left(\varphi ,\omega \right)}, \end{array}$$

where *g*_*x*_ and *g*_*y*_ can be computed as follows:

(9) $$\begin{array}{c} g_{x}\left(\varphi ,\omega \right)=I\left(\varphi +1,\omega \right)-I\left(\varphi -1,\omega \right), \end{array}$$

(10) $$\begin{array}{c} g_{y}\left(\varphi ,\omega \right)=I\left(\varphi ,\omega +1\right)-I\left(\varphi ,\omega -1\right), \end{array}$$

where *φ* and *ω* represent the coordinate position of a pixel in the picture. The [0-360] orientation is divided into *m* regions, and the pixels are divided into *m* regions to count HOG by gradient direction. Then, the HOG eigenvectors which contain *m* values have to be normalized by the following formula:

(11) $$\begin{array}{c} V=\frac{V}{\sqrt{{\left\|V\right\|}_{2}^{2}+\varepsilon^{2}}}, \end{array}$$

where *V* represents the HOG feature vector and *ε* is a small constant. Finally, HOG features of each spatial grid from all pyramid levels are concatenated to be PHOG feature descriptors. In this experiment, we set parameters *L* = 3 and *m* = 8. The number of grids in four levels is 85 (1 + 2 × 2 + 4 × 4 + 8 × 8), and converted the PSSM into a 680 (85 × 8) dimensional vector.  Figure 2 gives an example of merging HOG describers into PHOG describers.

### 2.5. Rotation Forest (RF)

Rodriguez et al. developed the early integrated forest into the rotation forest (RF) [33]. Rotation forest works well on difference promotions and classifications of small sample data sets [34]. In particular, the RF classifier has outstanding performance on balancing the diversity and accuracy of the base classifier by rotating the subsets. Meanwhile, the model also preserves the efficiency, interpretability, and simplicity of the decision tree. In this paper, we employ RF to predict DTIs. The detailed process is shown as follows.

In practical terms, the data is randomly separated into *K* subsets containing disjoint features. Afterward, the bootstrap and Principal Component Analysis (PCA) method are applied in subsets to obtain rotation matrices with high diversity. Finally, these matrixes are fed into the corresponding base classifier, and the scores of each decision tree are counted. The matrix *X* of *n* × *m* is treated as a training feature set which contains *m* features of *n* samples, and *T* = (*t*_1_, *t*_2_,⋯,*t*_*n*_)^*T*^ stores the corresponding labels of *n* samples. RF has *L* base classifiers *D*_*i*_. The detailed training process of the base classifier is as follows.

(I) After optimizing the model, the data set *M* is separated into *K* disjoint subsets at random, and each subset has *C* = *m*/*k* features
(II) Let *M*_*i*,*j*_ represent the *j*_th_ subset of *M*, and *X*_*i*,*j*_ is the corresponding feature set of *M*_*i*,*j*_. Then, calculate the new training feature set *X*_*i*,*j*_′ by bootstrap sampling on 75% of *X*_*i*,*j*_
(III) Perform PCA on *X*_*i*,*j*_′ to get the principal component coefficients which can be represented as *a*_*i*,*j*_^(1)^, *a*_*i*,*j*_^(2)^, ⋯*a*_*i*,*j*_^(*C*_*j*_)^
(IV) These coefficients construct the sparse rotation matrix Z_*i*_ as follows:
  (12) $$\begin{array}{c} Z_{i}=\left[\begin{array}{cccc} a_{i,1}^{\left(1\right)},a_{i,1}^{\left(2\right)},\cdots a_{i,1}^{\left(C_{1}\right)} & 0 & \cdots & 0\\ 0 & a_{i,2}^{\left(1\right)},a_{i,2}^{\left(2\right)},\cdots a_{i,2}^{\left(C_{2}\right)} & \cdots & 0\\ \vdots & \vdots & \ddots & \vdots \\ 0 & 0 & \cdots & a_{i,K}^{\left(1\right)},a_{i,K}^{\left(2\right)},\cdots a_{i,K}^{\left(C_{k}\right)} \end{array}\right]. \end{array}$$

In the process of classification, the possibility that sample *x* belongs to category *y*_*i*_ is *d*_*i*,*j*_(*x*Z_*i*_^*a*^) generated by base classifier *D*_*i*_. Subsequently, count the confidence degrees that *x* belongs to each class by mean combination as follows:

(13) $$\begin{array}{c} \mu_{j}\left(x\right)=\frac{1}{L}\overset{L}{\underset{i=1}{\sum }}d_{i,j}\left({xZ}_{i}^{a}\right). \end{array}$$

The sample *x* will be distributed into the most possible class in accordance with the degree.

## 3. Results and Discussion

### 3.1. Evaluation Criteria

Throughout the experiments, accuracy (Acc.), sensitivity (Sen.), precision (Pre.), specificity (Spec.), and Matthews correlation coefficient (MCC) comprehensively appraise the prediction performance. These criteria can be defined as follows:

(14) $$\begin{array}{c} \text{Acc}.=\frac{\text{TP}+\text{TN}}{\text{TP}+\text{FP}+\text{TN}+\text{FN}}, \end{array}$$

(15) $$\begin{array}{c} \text{Sen}.=\frac{\text{TP}}{\text{TP}+\text{TN}}, \end{array}$$

(16) $$\begin{array}{c} \text{Pre}.=\frac{\text{TP}}{\text{FP}+\text{TP}}, \end{array}$$

(17) $$\begin{array}{c} \text{Spec}.=\frac{\text{TN}}{\text{TP}+\text{FP}}, \end{array}$$

(18) $$\begin{array}{c} \text{MCC}=\frac{\text{TN}\times \text{TP}-\text{FN}\times \text{FP}}{\sqrt{\left(\text{TN}+\text{FN}\right)\times \left(\text{TP}\times \text{FP}\right)\times \left(\text{TN}+\text{FP}\right)\times \left(\text{TP}\times \text{FN}\right)}}, \end{array}$$

where true positive (TP) represents the sum of interacting drug-target pairs with correct predictions, true negative (TN) reflects the aggregate of noninteracting drug-target pairs with correct predictions, false positive (FP) denotes the count of noninteracting drug-target pairs with incorrect classifications, and false negative (FN) represents the count of interacting drug-target pairs with incorrect classifications. Furthermore, receiver operating characteristic (ROC) curves are employed to depict results [35], and the area under the curve (AUC) is calculated to justify the prediction feasibility [36].

### 3.2. Parameter Discussion

**Figure 3:**
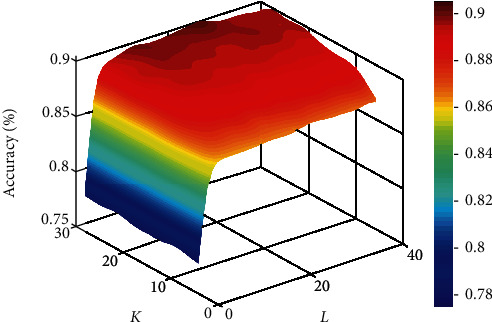
Accuracy surface of RF classifier influenced by parameters *K* and *L*.

In this experiment, parameters *K* and *L* are relevant to the results of the model. The *K* value and *L* value represent the numbers of the feature subsets and decision trees of RF, respectively. We applied the grid search algorithm to get the optimum parameters [37]. The method indicates that the accuracy ascends with the growth of the *L* value. When *K* = 28 and *L* = 26, the model has the best performance. Hence, we set the *K* value and the *L* value as 28 and 26, respectively.  Figure 3 shows the accuracy surface of the RF classifier influenced by parameters *K* and *L*.

### 3.3. Fivefold CV Results on Four Data Sets

This section applied 5-fold CV on *enzyme*, *ion channel*, *GPCR*, and *nuclear receptor* data sets to obtain evaluation results for further verifying the reliability of our model. During the validation, the data set was broken into five subsets on average. Specifically, each subset took turns to be regarded as the testing part; the other four subsets merged into the training part in five repetitive experiments. Tables 2–5 list the results of validations on benchmark data sets.

**Figure 4:**
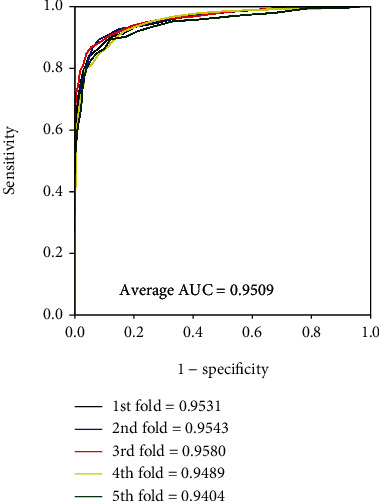
ROC curves performed by our approach on the *enzyme* data set.

**Figure 5:**
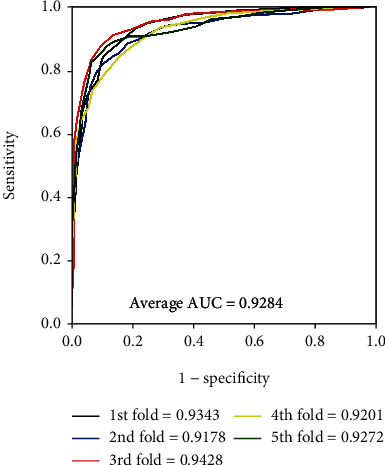
ROC curves performed by our approach on the *ion channel* data set.

**Figure 6:**
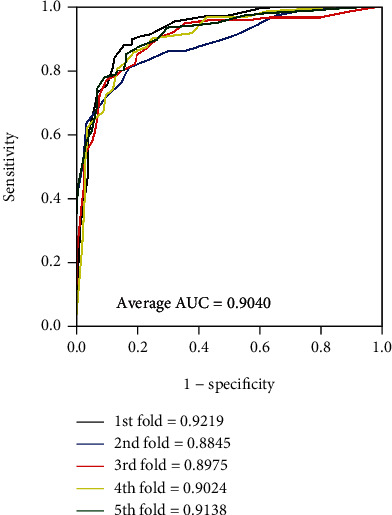
ROC curves performed by our approach on the *GPCRs* data set.

**Figure 7:**
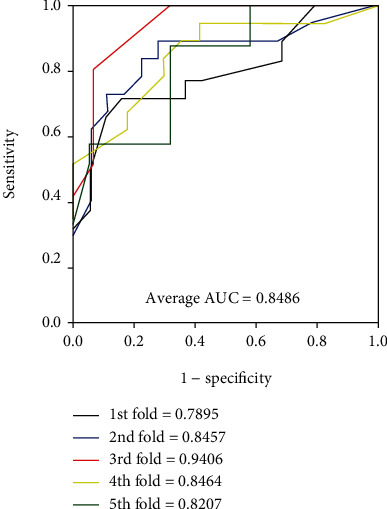
ROC curves performed by our approach on the *nuclear receptor* data set.

It is obvious that the model worked well on four golden standard data sets from Tables 2–5. In terms of the results yielded by the *enzyme* data set, the average accuracy, precision, sensitivity, specificity, and MCC are 88.96%, 89.76%, 87.92%, 90.01%, and 77.93% with standard deviations of 1.13%, 1.82%, 1.25%, 1.50%, and 2.29%, respectively. As for the results yielded on the *ion channel* data set, the accuracy, precision, sensitivity, specificity, and MCC come to be 86.37%, 86.24%, 86.45%, 86.24%, and 72.72% with standard deviations of 1.90%, 2.02%, 2.74%, 1.75%, and 3.84%, respectively. When performing the model on the *GPCR* data set, we obtained the average accuracy, precision, sensitivity, specificity, and MCC of 82.88%, 83.10%, 82.53%, 83.32%, and 65.78% with standard deviations of 1.49%, 3.30%, 3.26%, 2.24%, and 3.03%, respectively. When verifying the proposed model on the *nuclear receptor* data set, the model generates average accuracy, precision, sensitivity, specificity, and MCC of 76.92%, 74.45%, 82.97%, 71.04%, and 54.94% with standard deviations of 6.30%, 9.01%, 8.43%, 12.14%, and 10.91%, respectively. The difference between the sample quantities caused the gap of the evaluating criteria and standard deviations between four benchmark data sets. The average AUC of the proposed model were 0.9509, 0.9284, 0.9040, and 0.8486, respectively. Figures 4–7 give the ROC curves for the four benchmark data sets.

### 3.4. Comparison between the Models with PHOG Descriptor and LPQ Descriptor

**Figure 8:**
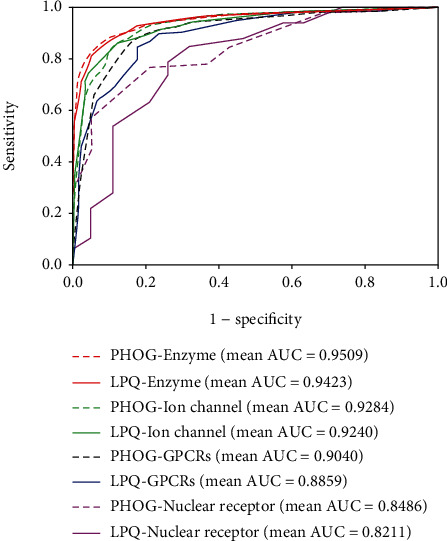
Performance comparison between LPQ and PHOG on four golden standard data sets.

For fairly evaluating the performance of the PHOG descriptor, we also conducted the experiments with local phase quantization (LPQ) which has a wide application prospect in spatial fuzzy image texture description processing for blurred-invariant property [38, 39].  Table 6 displays the comparison between PHOG and LPQ with rotation forest. The summarized table clearly indicates that the model with PHOG descriptors has a performance promotion than the LPQ descriptors on four golden standard data sets. In particular, the precision, sensitivity, specificity, and MCC all improved in the *ion channel* and *GPCR* data sets.  Figure 8 plots the ROC curves of the PHOG and LPQ models on four data sets with mean AUC values. As can be noted, the AUC values of the PHOG model are higher than the LPQ model. Especially in the *GPCR* and *nuclear receptor* data sets, AUC gaps attend to 1.81% and 2.75%, respectively. Hence, our model can effectively describe PSSM to identify potential interacting drug-target pairs.

### 3.5. Comparison with Other Classifiers

At present, the classifiers which were used in predicting DTIs are mainly based on traditional machine learning methods. In this section, we adopted advanced SVM and LGBM to combine PHOG descriptors. In the rotation forest, we set parameters *K* = 28 and *L* = 26 by utilizing the grid search method. After parameter optimization, SVM embedded Gaussian kernel function with parameters *C* = 0.2 and Gamma = 40. Parameter *C* prevents SVM from over fitting, and Gamma determines the number of support vectors. LGBM is based on a gradient boosting framework, and it is widely used in classification in industrial practice for it is time-saving and memory-conserving. After conducting grid method searching, the best results can be obtained by setting the number of leaves to 60, the learning rate to 0.05, and the number of training rounds to 40.

**Figure 9:**
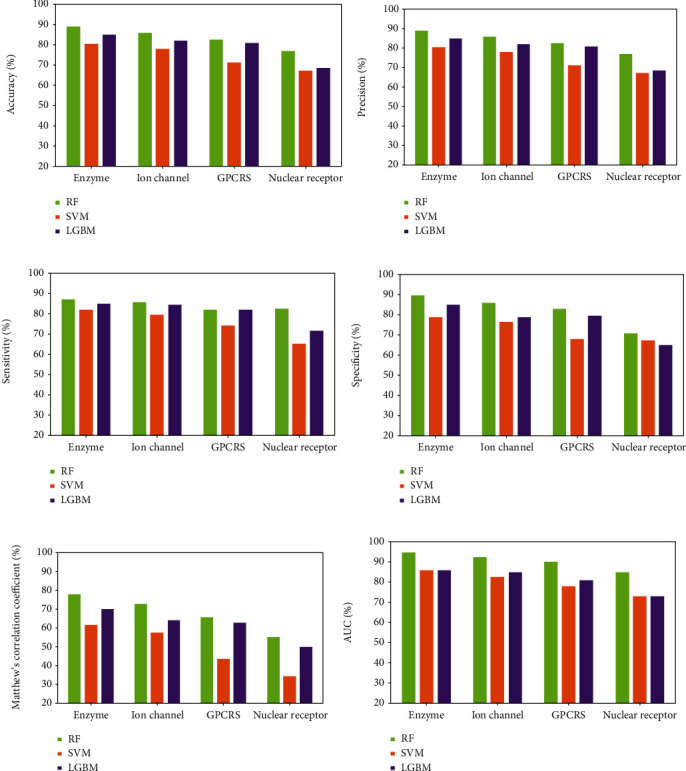
Comparison of experimental outcomes of RF, SVM, and LGBM on four benchmark data sets with six evaluating indicators: (a) accuracy; (b) precision; (c) sensitivity; (d) specificity; (e) MCC; (f) AUC.

**Figure 10:**
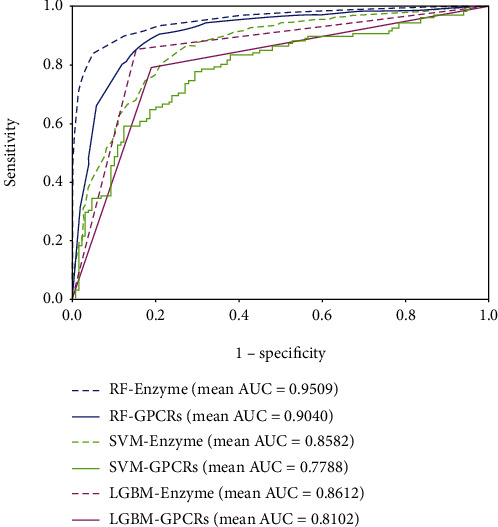
ROC curves performed by RF, SVM, and LGBM on the *enzyme* and *GPCR* data sets.

**Figure 11:**
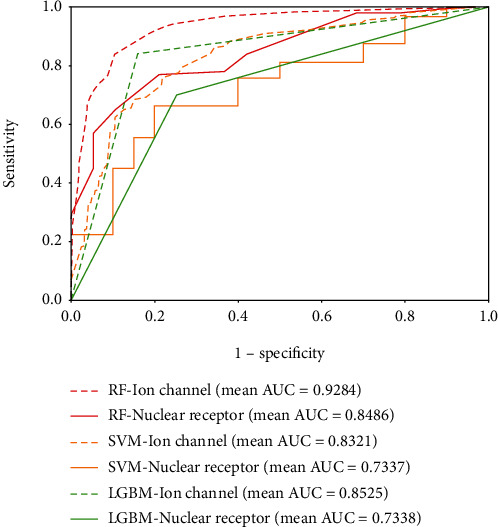
ROC curves performed by RF, SVM, and LGBM on the *ion channel* and *nuclear receptor* data sets.

Figure 9 gives the results of RF, SVM, and LGBM on the *enzyme*, *ion channel*, *GPCR*, and *nuclear receptor* data sets, and it clearly reports that RF has a better performance with the PHOG descriptor than the other classifiers on verifying interacting drug-target pairs. The mean accuracy of RF is 8.30%, 8.07%, 11.43%, and 9.56% higher than SVM on the four golden standard data sets. Compared with the LGBM algorithm, the accuracy of RF improved 3.90%, 4.34%, 1.65%, and 8.33%, respectively. Figures 10 and 11 depict the ROC curves of the benchmark data sets generated by the rates of true positive (TP) and false positive (FP). In addition, mean AUC values are also attached to each graph for more intuitively describing the effect of different classifiers. The reliability of predicting DTIs of the model is proportional to the value of AUC. It can be observed that RF has performance promotions of 9.27%, 9.63%, 12.52%, and 11.49% against SVM on the four benchmark data sets. The value gaps of AUC between RF and LGBM are 8.97%, 7.59%, 9.38%, and 11.48%, respectively, on the four data sets. Accordingly, RF is more competitive than the other models in predicting DTIs.

### 3.6. Comparison with Other Methods

To date, many researchers have innovatively provided effective solutions for the prediction of DTIs. In order to further validate the efficiency of our model, we selected such previous models as MLCLE [40], NetCBP [41], SIMCOMP [42], WNN-GIP [43], AM-PSSM [44], NetLapRLS [45], MSCMF [20], and Bigram-PSSM [46] to analyze the performance of the proposed model. Meanwhile, all of these models are under the 5-fold CV framework on benchmark data sets. The average AUC values of these methods obviously indicate that the effect of our model has a significant enhancement in prediction in Table 7. In terms of the *enzyme*, *ion channel*, and *GPCR* data sets, the growths of the AUC reached 0.0029, 0.0119, and 0.0320, respectively. With regard to the *nuclear receptor* data set, Bigram-PSSM has the best performance with an AUC improvement of 0.0204 than our model. The results illustrate that the model which embeds PHOG descriptors and rotation forest is competent to effectively identify DTIs.

## 4. Conclusion

In this paper, we fused the pyramid histogram of oriented gradients (PHOG), Position-Specific Scoring Matrix (PSSM), and rotation forest (RF) into a novel computational model to predict the interactions between drugs and targets. To prove the reliability of the proposed model, a series of experiments have been conducted. Specifically, we first altered the feature extraction method of the proposed model with LPQ to assess the feature description ability of PHOG. This paper also experimented on state-of-the-art LGBM and SVM with the same features to validate the performance of RF. Among them, the proposed model achieves mean accuracies of 88.96%, 86.37%, 82.88%, and 76.92% on the *enzyme*, *ion channel*, *G protein-coupled receptor* (*GPCR*), and *nuclear receptor* data sets. The results obviously illustrate that the PHOG features can trace the local characteristics and assist the model to improve the accuracy even compared with LPQ. Meanwhile, the model is considered to be an extraordinarily suitable tool for providing candidates of drug discovery. In the subsequent work, we will experiment with more methods to further raise the feasibility of the prediction model.

## 5. Limitation and Future Work

Although the model shows an improved prediction ability than other models, we still noticed the singleness of the local feature, and the noise existing in features also has an adverse effect on forming describers. The main limitations of the model can be explained from two aspects. On the one side, the utilized feature extraction method is sensitive to local feature information. However, it is hard to excavate the global feature information of samples. On another side, the same number of unlabelled samples is randomly selected to be negative samples as the known interacting drug-target pairs; hence, the model wastes a large number of unselected negative samples. The feature studies will mainly focus on the processes of feature extraction and classification. The external edge features which have an excellent application prospect in the field of image tamper prevention will integrate the internal features to comprehensively describe bioinformation with less noise. Meanwhile, unsupervised learning models will be adopted to confront the waste of data sets, and it will make full use of high-throughput unbalanced data. These improvements will bring new challenges and opportunities to develop robust prediction tools for enhancing the model prediction accuracy.

## Figures and Tables

**Table 1:** The statistical quantities for drugs, target proteins, and DTIs.

Data set	Drugs	Target proteins	Interactions
Enzyme	445	664	2926
Ion channel	210	204	1467
GPCRs	223	95	635
Nuclear receptor	54	26	90

**Table 2:** 5-fold CV performance of our approach on the *enzyme* data set.

Test set	Acc. (%)	Pre. (%)	Sen. (%)	Spec. (%)	MCC (%)
1	88.89	88.31	88.93	88.85	77.77
2	90.34	91.85	89.27	91.49	80.71
3	89.83	91.61	88.07	91.65	79.73
4	87.69	88.19	86.99	88.40	75.39
5	88.03	88.83	86.34	89.65	76.07
Average	88.96 ± 1.13	89.76 ± 1.82	87.92 ± 1.25	90.01 ± 1.50	77.93 ± 2.29

**Table 3:** 5-fold CV performance of our approach on the *ion channel* data set.

Test set	Acc. (%)	Pre. (%)	Sen. (%)	Spec. (%)	MCC (%)
1	86.44	86.13	87.83	84.97	72.85
2	84.74	84.67	85.23	84.25	69.49
3	88.64	87.95	89.46	87.84	77.30
4	84.24	83.88	82.37	85.90	68.35
5	87.80	88.55	87.38	88.24	75.60
Average	86.37 ± 1.90	86.24 ± 2.02	86.45 ± 2.74	86.24 ± 1.75	72.72 ± 3.84

**Table 4:** 5-fold CV performance of our approach on the *GPCR* data set.

Test set	Acc. (%)	Pre. (%)	Sen. (%)	Spec. (%)	MCC (%)
1	85.04	82.96	88.19	81.89	70.22
2	81.89	84.85	81.16	82.76	63.73
3	81.50	77.50	82.30	80.85	62.86
4	83.79	84.35	80.83	86.47	67.49
5	82.21	85.83	80.15	84.62	64.58
Average	82.88 ± 1.49	83.10 ± 3.30	82.53 ± 3.26	83.32 ± 2.24	65.78 ± 3.03

**Table 5:** 5-fold CV performance of our approach on the *nuclear receptor* data set.

Test set	Acc. (%)	Pre. (%)	Sen. (%)	Spec. (%)	MCC (%)
1	75.00	75.12	70.59	78.95	49.77
2	77.78	77.78	77.89	77.65	55.56
3	86.11	85.71	90.00	81.25	71.81
4	77.14	72.73	88.89	64.71	55.44
5	68.57	60.90	87.50	52.63	42.12
Average	76.92 ± 6.30	74.45 ± 9.01	82.97 ± 8.43	71.04 ± 12.14	54.94 ± 10.91

**Table 6:** The comparison between LPQ and PHOG with rotation forest in terms of accuracy (Acc.), precision (Pre.), sensitivity (Sen.), specificity (Spec.), and Matthews correlation coefficient (MCC) on four types of benchmark data sets.

Data set	Method	Acc. (%)	Prec. (%)	Sen. (%)	Spec. (%)	MCC (%)
Enzyme	LPQ+RF	88.48 ± 0.87	90.30 ± 1.92	87.10 ± 1.52	90.65 ± 1.87	77.79 ± 1.76
	PHOG+RF	88.96 ± 1.13	89.76 ± 1.82	87.92 ± 1.25	90.01 ± 1.50	77.93 ± 2.29
Ion Channel	LPQ+RF	85.36 ± 1.00	85.22 ± 1.84	85.53 ± 1.37	85.13 ± 2.15	70.70 ± 2.00
	PHOG+RF	86.37 ± 1.90	86.24 ± 2.02	86.45 ± 2.74	86.24 ± 1.75	72.72 ± 3.84
GPCRs	LPQ+RF	82.02 ± 2.82	81.59 ± 5.65	82.34 ± 3.43	81.62 ± 4.39	63.92 ± 5.56
	PHOG+RF	82.88 ± 1.49	83.10 ± 3.30	82.53 ± 3.26	83.32 ± 2.24	65.78 ± 3.03
Nuclear receptor	LPQ+RF	75.78 ± 6.78	75.66 ± 6.80	77.62 ± 6.17	72.93 ± 14.28	50.89 ± 15.46
	PHOG+RF	76.92 ± 6.30	74.45 ± 9.01	82.97 ± 8.43	71.04 ± 12.14	54.94 ± 10.91

**Table 7:** The comparison of AUC values obtained by the proposed model and other advanced model on four benchmark data sets.

Model	Enzyme	Ion channel	GPCRs	Nuclear receptor
MLCLE	0.842	0.795	0.850	0.790
NetCBP	0.8251	0.8034	0.8235	0.8394
SIMCOMP	0.863	0.776	0.867	0.856
WNN-GIP	0.861	0.775	0.872	0.839
AM-PSSM	0.843	0.722	0.839	0.767
NetLapRLS	0.9013	0.9165	0.7711	0.6772
MSCMF	0.9142	0.776	0.867	0.856
Bigram-PSSM	0.948	0.889	0.872	0.869
Our method	0.9509	0.9284	0.9040	0.8486

## Data Availability

The data are original, and the data source is restricted.
